# Comparison of follicular dynamics, superovulatory response, and embryo recovery between estradiol based and conventional superstimulation protocol in buffaloes (*Bubalus bubalis*)

**DOI:** 10.14202/vetworld.2015.983-988

**Published:** 2015-08-12

**Authors:** Narinder Singh, G. S. Dhaliwal, V. S. Malik, D. Dadarwal, M. Honparkhe, S. Singhal, P. S. Brar

**Affiliations:** 1 Department of Veterinary Gynaecology and Obstetrics, Guru Angad Dev Veterinary and Animal Sciences University, Ludhiana, Punjab, India; 2 Department of Teaching Veterinary Clinical Complex, Guru Angad Dev Veterinary and Animal Sciences University, Ludhiana, Punjab, India; 3 Large Animal Clinical Sciences, Western College of Veterinary Medicine, University of Saskatchewan, Saskatoon, Canada

**Keywords:** buffalo, embryo, estradiol-17β, superstimulation, ovulation rate

## Abstract

**Aim:**

To evaluate the follicular dynamics, superovulatory response, and embryo recovery following superstimulatory treatment initiated at estradiol-17β induced follicular wave emergence and its comparison with conventional superstimulatory protocol in buffaloes.

**Materials and Methods:**

Six normal cycling pluriparous buffaloes, lactating, 90-180 days post-partum, and weighing between 500 and 660 kg were superstimulated twice with a withdrawal period of 35 days in between two treatments. In superstimulation protocol-1 (estradiol group) buffaloes were administered estradiol-17β (2 mg, i.m.) and eazibreed controlled internal drug release (CIDR) was inserted intravaginally (day=0) at the random stage of the estrous cycle. On the day 4, buffaloes were superstimulated using follicle stimulating hormone (FSH) 400 mg, divided into 10 tapering doses given at 12 hourly intervals. Prostaglandin F2α analogs (PGF2α) was administered at day 7.5 and day 8, and CIDR was removed with the second PGF2α injection. In superstimulation protocol - 2 (conventional group) buffaloes were superstimulated on the 10^th^ day of the estrous cycle with same FSH dose regimen and similar timings for PGF2α injections. In both groups, half of the buffaloes were treated with luteinizing hormone (LH) 25 mg and other half with 100 ug buserelin; gonadotrophin releasing hormone (GnRH) analog at 12 h after the end of FSH treatment. All buffaloes in both protocols were inseminated twice at 12 and 24 h of LH/GnRH treatment. Daily ultrasonography was performed to record the size and number of follicles and superovulatory response.

**Results:**

Significantly higher number of small follicles (<8 mm) was present at the time of initiation of superstimulatory treatment in the estradiol group compared to the conventional group (12.5±0.80 vs. 7.3±1.21, respectively, p=0.019), however, the number of ovulatory size follicles (≥8 mm) did not differ significantly between the respective groups (15.5±1.24 vs. 12.2±1.30; p=0.054). Total embryos and transferable embryos recovered were non-significantly higher in the estradiol group compared to the conventional group (5.83±0.86 vs. 4.67±1.16, p=0.328, and 3.67±0.93 vs. 2.67±0.68, p=0.437, respectively). The significant higher proportion of transferable embryos were recovered in buffaloes treated with LH compared to GnRH (73.3% vs. 48.5%; p=0.044).

**Conclusion:**

The average number of ovulatory size follicles (>8 mm), corpora lutea, and transferable embryos was higher in buffaloes superstimulated at estradiol-induced follicular wave compared to the conventional protocol: Further the percentage of transferable embryos was significantly higher in buffaloes administered with LH compared to GnRH.

## Introduction

Buffalo is the mainstay of Indian dairy industry contributing more than 50% of milk to national milk pool which is much higher than the contribution of almost double the population of cattle [1]. However, the productivity of buffalo is low and is significantly hampered by various reproductive factors namely late attainment of puberty, poor estrus expression, seasonality, and post-partum anestrus. Multiple ovulation and embryo transfer (MOET) involving superstimulation and *in vivo* embryo collection have the potential to increase the contribution of superior females buffaloes to the gene pool of the population and it has been successfully used for faster dissemination of superior germplasm through the production of breeding sires from superior females in cattle [2,3]. In buffaloes, the application of MOET is limited due to variable superovulatory response and low yield of (<1) transferable embryos [4,5]. Similar to cattle, Misra [6] reported that over 26% superstimulated buffaloes produced ≥6 embryos per collection and *in vivo* embryo production could be a viable option in buffaloes on the identification of good donors.

So far conventional superstimulatory protocols have been used in buffaloes involving the initiation of superstimulatory treatment during ­mid-cycle (8-12^th^ day), to coincide with the approximate time of emergence of the second follicular wave. It has been observed that asynchrony of even 1 day between wave emergence and initiation of superstimulatory treatment may significantly reduce the superovulatory response [7]. Therefore, accurate estrus detection followed by initiation of superstimulatory treatment coinciding with the emergence of the second follicular wave is the key factors to achieve optimal results. Detection of estrus is difficult in buffaloes and the emergence of second follicular wave may also differ depending upon the estrous cycle being two- or three-wave affecting the initiation of superstimulatory treatment at emergence of second follicular wave in conventional protocols. Further, the presence of dominant follicle is reported to have an inhibitory effect on superstimulation that means nearly 80% period of the estrous cycle is not conducive to initiate superstimulatory treatment. Beg *et al*. [8] also pointed out that the standardizing the superovulatory schemes in the middle of the estrus cycle are more difficult in buffaloes compared to cattle.

To obviate these problems, an alternative approach could be to develop protocols which eliminate the need of estrus detection and necessary waiting until mid-cycle for initiating the superstimulatory treatment and permit fixed timed insemination of donors. In cattle, this has been successfully achieved following the induction of new follicular wave by removing dominant follicle through mechanical (follicle ablation) or hormonal approaches (reviewed in [9]). In buffaloes, few attempts have been made to apply such protocols with limited success [10]. However, no comparative study between initiation of superstimulatory protocol on exogenously controlled follicular wave emergence and conventional protocol is available in buffaloes. The emergence of follicular wave can be controlled in buffaloes through the administration of 1.5 mg estradiol-17β at a random stage of the estrous cycle [11] which could facilitate recruitment of a pool of growing follicles of uniform size on superstimulation. Therefore, the study was designed to assess the impact of initiating superstimulatory treatment at the beginning of estradiol-17β-induced follicular wave emergence on follicular dynamics, superovulatory response, and viable embryo recovery and its comparison with the conventional protocol in buffaloes.

## Materials and Methods

### Ethical approval

The present study being a part of a larger study for doctorate thesis, approval had been obtained from the Institutional Animal Ethics Committee. The embryo collection was done as per standard procedure without harming the animals.

### Treatment groups

Six normal cycling healthy buffaloes, with average body weight 500 - 660 kg, 90-180 days post-partum, superstimulated twice with a withdrawal period of 35 days in between two treatments were used for the study.

In Group I (estradiol group, n=6): Buffaloes were administered estradiol-17β (2 mg, i.m.) along with insertion of eazibreed controlled internal drug release (CIDR) (designated as day=0), the superstimulatory treatment was initiated on the 4^th^ day (day of follicular wave emergence) using follicle stimulating hormone (FSH) 400 mg NIH-FSH-P1, given daily intramuscularly in divided, tapering doses (60, 60, 50, 50, 40, 40, 30, 30, 20, 20 mg), over a period of 5 days. All the buffaloes were given two injections of prostaglandin F2α analog (PGF2α) cloprostenol 500 μg at 84 and 96 h after the start of superstimulatory treatment and CIDR was removed at the time of second prostaglandin injection.

Group II conventional superstimulation protocol: Buffaloes (n=6) were superstimulated as per the conventional protocol by initiating the superstimulatory treatment on the 10^th^ day of induced estrus using same dose regimen of FSH as above. Similarly, buffaloes were given two injections of PGF2α (500 μg cloprostenol; i/m) at 84 and 96 h after the start of superstimulatory treatment.

In both Groups I and II, half of the buffaloes were administered 25 mg luteinizing hormone (LH), and half were administered 100 ug buserelin (gonadotrophin releasing hormone (GnRH) analog) at 12 h after the end of superstimulatory treatment. The fixed time insemination was done at 12 and 24 h of LH/GnRH treatment.

### Observations

Ultrasonography was carried out daily using ultrasound machine (Mindray, China) equipped with B-mode linear array trans-rectal probe (7.5 MHz) from the start of experiment till the day of embryo recovery to record the size and the number of follicles followed with number of ovulations and luteal dynamics. Embryos were collected by flushing the uterus of the donor animals non-surgically on day 5.5 using two-way Worrlien catheter using flushing media - Dulbecoo’s phosphate buffered saline supplemented with 0.4% bovine serum albumin and antibiotics at standard rates. Embryos were searched and evaluated morphologically using zoom stereomicroscope and were graded as per the manual of International Embryo Transfer Society [12].

### Statistical analysis

The data are presented as means and standard errors for all variables. After confirming the normality of data and homogeneity of variance, Student’s *t*-test (two-tailed) was applied to compare mean values of treatments in SPSS-16 Statistical program. A p=0.05 was considered as significant.

## Results

All the buffaloes responded to superstimulatory treatment by developing multiple corpora lutea (CLs) on the ovaries. The results are presented in Table-1. Significantly higher number of small follicles (<8 mm) was present at the time of initiation of superstimulatory treatment in the estradiol group compared to the conventional group (12.5±0.80 vs. 7.3±1.21, respectively, p=0.019), however, during estrus the number of ovulatory size follicles (≥8 mm) did not differ significantly between the respective groups (15.5±1.24 vs. 12.2±1.30; p=0.054). The average number of follicles ≥8 mm size were higher at the time of initiation of superstimulatory treatment during mid-cycle in the conventional group compared to the estradiol group (0.83±0.32 vs. 0.17±0.20; p=0.102).

**Table-1:** Follicular dynamics and embryo production in buffalo’s superstimulated using the estradiol based and the conventional superstimulation protocol in buffaloes.

Parameters	Estradiol based protocol (n=6)	Conventional protocol (n=6)
At start of superstimulation protocol		
Number of follicles <8 mm	12.50±0.80*	7.33±1.21*
Number of follicles ≥8 mm	0.17±0.20	0.83±0.32
On the day of estrus		
Number of follicles <8 mm	2.67±1.12	3.50±0.75
Number of follicles ≥8 mm	15.50±1.24	12.17±1.30
Total number of CLs (day 5 post AI)	12.17±1.36	10.33±0.63
Ovulation rate (number of ovulatory size follicles/number of CLs)	78.49	84.9
Number of anovulatory follicles≥8 mm (day 5 post AI)	2±1.75	1±0.60
Number of embryos+ova recovered	5.83±0.86	4.67±1.16
Recovery rate (number of embryo+ova recovered/number of CLs)	47.95	45.16
Transferable embryos	3.67±0.93	2.67±0.68
Percent transferable embryos	62.86	57.14

Values marked with superscript (*) in a row differ significantly at 5% level, CLs=Corpora lutea, AI=Artificial insemination

No significant differences were observed in percent ovulation (78.5% vs. 84.9%, p=0.289), the number of CLs on day 5 post artificial insemination (AI) (12.2±1.36 vs. 10.3±0.63, p=0.280), embryo recovery rate (47.95% vs. 45.16%), and total number of recovered embryos (5.83±0.86 vs. 4.67±1.16, p=0.328) in the estradiol group compared to the conventional group, respectively. Further, a mean number of transferable embryos recovered were non-significantly higher in the estradiol group compared to the conventional group (3.67±0.93 vs. 2.67±0.68, p=0.437).

The administration of LH or GnRH did not have any significant effect on ovulation rate and a number of embryos recovered in buffaloes (Table-2). However, the proportion of transferable embryos collected from buffaloes treated with LH were significantly higher in LH group compared to GnRH group (73.3% vs. 48.5%; p=0.044) having lower mean number of anovulatory follicles (1.±0.63 vs. 2.0±1.62) on the day of flushing.

**Table-2:** Effect of LH and GnRH on ovulation, embryo recovery, and the number of transferable embryos in buffaloes.

Parameters	LH (n=6)	GnRH (n=6)
Number of follicles on day of estrus		
Number of follicles <8 mm	3.17±0.91	3.0±0.80
Number of follicles ≥8 mm	14.50±0.92	13.17±1.68
Number of CLs (day 5 post AI)	12.0±1.21	10.50±0.55
Ovulation rate (%)	82.7	79.7
Number of anovulatory follicles ≥8 mm (day 5 post AI)	1.0±0.63	2.0±1.62
Number of embryos+ova recovered	5±1.24	5.50±0.93
Recovery rate (%)	41.7	52.4
Number of transferable embryos	3.67±0.76	2.67±0.73
Percent transferable embryos	73.3*	48.5*

Values marked with superscript (*) in a row differ significantly at 5% level, CLs=Corpora lutea, LH=Luteinizing hormone, GnRH=Gonadotropin releasing hormone, AI=Artificial insemination

## Discussion

*In vivo* embryo production could be a viable option in buffaloes on the identification of good donors. However, the detection of estrus is more difficult in buffaloes than cattle which made implementation of the conventional superstimulatory protocols more difficult in buffaloes resulting in poor success rates. The initiation of superstimulatory treatment following exogenous control of follicular wave emergence using estradiol-17β could eliminate the need for estrus detection and avoid the unnecessary waiting period until mid-cycle to initiate the conventional protocol. Therefore, the present work was undertaken to study the follicular dynamics, superovulatory response, embryo recovery following superstimulation protocol initiated at the beginning of estradiol-17β-induced follicular wave emergence and its comparison with the conventional protocol in buffaloes.

### Follicular dynamics

The number of small follicles (≤8 mm) present at the time of initiation of superstimulatory treatment was significantly higher in the estradiol group compared to the conventional group (Table-1). Similarly, following the treatment, a higher number of follicles reached ovulatory size (≥8 mm) in the estradiol group (p=0.054) compared to the conventional group on the day of estrus. The average number of follicles reaching ovulatory size was over 12 and 15 in the conventional and the estradiol groups, respectively. Our results corroborated the findings of Baruselli *et al*. [13] and Carvalho *et al*. [14] who showed that a sufficient number of follicles reach ovulatory size. A higher number of follicles reaching an ovulatory size in superstimulated buffaloes using FSH alone or FSH + pregnant mare’s serum gonadotropin had been reported by Abd-Allah *et al*. [15]. However, the earlier studies had used mainly conventional protocols for superstimulation and obtained a poor response in buffaloes. These studies pointed out the low population of ovarian primordial follicles in buffaloes compared to cattle as the possible cause for low superovultory response in buffaloes [4,16]. However, Azawi *et al*. [17] and Campanile *et al*. [18] reported the recruitment of a similar number of ovarian follicles in buffaloes and cattle during wave emergence.

The reason for higher number of follicles reaching ovulatory size (≥8 mm) in the estradiol group compared to the conventional group in the present study could be emergence of a new follicular wave 4 days later following administration of estradiol-17β [10] which led to availability of significantly higher number of smaller follicles of uniform size to undergo superstimulation. The lower number of the small follicle at the initiation of superstimulatory treatment could be due to asynchrony of the emergence of second follicular wave and initiation of superstimulatory treatment during mid-cycle in the conventional group compared to the estradiol group. In addition to that the presence of dominant follicles of ≥8 mm size (p=0.102) in three buffaloes at the beginning of superstimulatory treatment might have exerted an inhibitory effect on the growth of subordinate follicles in the conventional group.

### Ovulation rate

In general, the both groups showed higher ovulation rate (74% and 84%) in the present study. The similar high ovulation rate in superstimulated buffaloes has been reported by Lipinski *et al*. [19]. The moderate ovulation rate (~60%) and number of CLs (~9 CL) in buffaloes have been reported by Baruselli *et al*. [13]. However, the superstimulation studies carried out in 90s, the ovulation rate was much lower and ranged between 2 and 4 ovulations in buffaloes [20,21]. Patel *et al*. [22] also reported fewer ovulations (~4) following superstimulation in pandharpuri buffaloes.

The superovulatory response in terms of number of CLs on the 5^th^ day post AI in both groups was higher than reported by Misra and Joshi [20] and Agarwal *et al*. [21]. Misra [6] in a study reported that 12% of the superstimulated buffaloes developed >10 CLs. The higher number of CLs in both groups in this study could be due to the better synchrony of beginning of superstimulatory treatment and wave emergence in the estradiol group. In the conventional group, it could be due to better detection and induction of estrus (day=0) than previous studies which did not use ultrasonography to establish a day of estrus. The use of 5-day protocol over the 4 days superstimulatory protocol and administration of LH or GnRH for facilitating the process of ovulation could be another reason for higher ovulation and the number of CLs in the present study. It was observed that use of 5-day FSH protocol gave better results compared to 4 days dosage regimen.

The comparatively higher number of CLs in the estradiol group than the conventional group on the 5^th^ day post AI could be due to the higher number of follicles reaching ovulatory size (≥8 mm) in the estradiol group compared to the conventional group. The presence of higher number of dominant follicles at the initiation of superstimulatory protocol in the conventional group could be another reason. The decreased superovulatory response due to the presence of dominant follicle has also been reported by others [23]. Li *et al*. [24] reported that the immunoneutralization against inhibin or follistatin was effective in enhancing ovarian follicular development, ovulation rate, and a number of embryos recovered from superstimulated buffaloes.

### Embryo recovery

The moderate embryo recovery rate of ~45-48 was observed in the present study. The recovery rate achieved in the present study was higher than (~34%) reported by Baruselli *et al*. [13] and Carvalho *et al*. [14] but lower than 63-80% reported in cattle. Misra [6] also reported recovery rate of >60% and observed that recovery decreased to 33% when a number of ovulations were more than 15 per buffalo donor. It was proposed that lower embryo recovery in buffaloes could be due to changed uterine environment as a result of high estrogen level [25], failure of oocyte capture and/or of oocyte transport along the oviduct [13], fragile connection between the oocyte and granulosa cells [26] higher number of anovulatory follicles, a more rigid ovary-mesovarium connection, and a thicker infundibulum muscle layer than cattle [27,28], however none of these aspects has been critically studied. The effort to improve embryo recovery by giving progesterone during the periovulatory period [29] was unsuccessful due to the overall failure of ovulation. The use of recombinant bovine somatotropin to improve fragile connections between oocyte and granulosa cells also proved inconclusive [14]. The exact cause of this low efficiency in embryo recovery is still unknown.

The average number of embryos recovered in the present study was higher in the estradiol group than the conventional group. The two studies in the literature have reported the recovery of similar or higher number of embryos. Misra *et al*. [30] have recovered up to mean of 7.5 embryos from 10 superstimulated buffaloes in India and Jiang *et al*. [31] reported the recovery of average six embryos per superovulation in China. In another study, Wang *et al*. [32] reported the higher recovery of eight embryos per animal after sacrifice with a recovery rate of 56.5%, out of which seven were of transferable grade. Few other studies involving immunoneutralization against inhibin or follistatin [24] and administration of PGF2α during the periovulatory period [33] has also found effective in enhancing a number of embryos recovered from superstimulated buffaloes.

### Transferable embryos

The average number of transferable embryos recorded in the estradiol group was non-significantly higher than the conventional group. Misra *et al*. [34] reported an average recovery of 4.2 total embryos and 2.1 viable embryos per flushing using purified FSH-P1 preparation. In isolated trials transferable embryos as high as 5.9 [30], >4 transferable embryos [31] and 7 transferable embryos after sacrificing [32] has also been achieved. The average number of transferable embryos in our study was higher than many other studies reporting only one to two transferable embryos in buffaloes [4,16,20,21]. The percentage transferable embryos achieved in both groups were comparable to Carvalho *et al*. [14], Heleil and El-Deeb [23] and lower than reported by Soares *et al*. [33] achieved using protocol involving administration of PGF2α during periovulatory period.

### Effect of LH and GnRH on superovulatory response

Differences in superovulatory response, embryo recovery and a number of transferable embryos were non-significant between buffaloes administered LH or GnRH in the present study (Table-2). However, the percentage of transferable embryos was significantly higher in buffaloes treated with LH (p=0.044) compared to GnRH group. The high estradiol level due to the presence of higher mean value of anovulatory follicles (2.0 vs. 1.0) on the day of embryo collection might be responsible for a lower percentage of transferable embryos in buffaloes administered with GnRH. Beg *et al*. [8] reported that buffaloes were more sensitive to higher estradiol levels than bovine which might have affected their uterine environment during superstimulation [25].

Techakumphu *et al*. [35] reported that supplementation of GnRH at 8-12 h after standing heat seemed to produce more transferable embryos than those treated at standing heat or the controls. Zicarelli *et al*. [36] did not find any significant differences in ovarian follicular response in buffaloes treated with GnRH agonist LH protocol (involving administration of LH as an ovulation inducer) compared to the conventional protocol. However, Carvalho *et al*. [10] reported that the GnRH agonist LH protocol was effective in improving ovulation rate in superstimulated buffaloes. Qin *et al*. [37] observed higher ovulation rate in buffaloes administered luteinizing hormone releasing hormone-A3 (GnRH) compared to LH.

## Conclusion

Significantly higher number of growing follicles was present in the estradiol group at the initiation of superstimulatory treatment compared to the conventional group. Superovulatory response in terms of number of CLs, total, and transferable embryos tend to be higher in the estradiol group compared to the conventional protocol indicating that the estradiol based protocol could be successfully used for superstimulation in buffaloes. In addition to this, the administration of LH in buffaloes subjected to fix timed AI following superstimulation produced a significantly higher percentage of transferable embryos compared to administration of GnRH. Therefore, the results of the present study indicated that initiation of superstimulatory treatment subsequent to the synchronization of follicular wave emergence by estradiol-17β and fixed timed AI could be successfully used for embryo production in buffaloes.

## Authors’ Contributions

The present study was part of NS’s PhD dissertation. The work was designed by GSD and PSB. The execution of experimentation protocol and performing ultrasound scanning was done by NS, VSM, and MH. The lab work was performed by NS, SS, and DD. Statistical analysis and drafting of the manuscript were done by NS, GSD, and DD. All authors read and approved the final manuscript.
